# Prevalence and Predictors of Dysmenorrhea, Its Effect, and Coping Mechanisms among Adolescents in Shai Osudoku District, Ghana

**DOI:** 10.1155/2019/5834159

**Published:** 2019-05-20

**Authors:** Kwabena Acheampong, Dorothy Baffour-Awuah, Daniel Ganu, Stalla Appiah, Xionfeng Pan, Atipatsa Kaminga, Aizhong Liu

**Affiliations:** ^1^Department of Epidemiology and Health Statistics, Xiangya School of Public Central South University, Changsha, Hunan 410078, China; ^2^Department of Public Health, Adventist University of Africa, Ongata Rongai 00503, Kenya; ^3^Departments of Nursing, Valley View University, Oyibi, Accra 00233, Ghana

## Abstract

**Background:**

Dysmenorrhea has been the most common gynecological problem worldwide. Reports of dysmenorrhea are greatest among individuals in their late teens and 20s and usually declining with age. It has also been reported that dysmenorrhea affects more than 80% of women in the reproductive age. The study objective was to examine the predictors of dysmenorrhea, its effect, and coping mechanisms among adolescents in Shai Osudoku District, Ghana.

**Methods:**

We conducted a cross-sectional study in September and November 2017 in selected schools in Shai Osudoku District, Ghana. We employed self-administered questionnaire to obtain data from adolescents volunteered to participate in the study. We analyzed the data using the SPSS programme IBM version 20. We used the Pearson chi-square test and multiple logistic regression analysis to assess the association between exposure variables and the outcome variable. The odds ratio was reported to establish the risk of dysmenorrhea at a confidence interval of 95%, and statistical significance was assumed at *p* < 0.05.

**Results:**

The prevalence of dysmenorrhea was 68.1% (95% CI, 65.0–72.0) with one-third recounting their pain as severe. The pain during menstruation negatively influences the daily physical activities (22.5%), school attendance (6.9%), concentration during classes' hours (27.9%), and academic performance (31.1%) of the respondents. Besides, adolescents who do not live with their parent experienced a 53.1% increase in odds of self-reporting dysmenorrhea (AOR, 1.53 (95% CI, 1.02–2.23)). Similarly, respondents who had irregular menstrual cycle experienced a 72.5% increase in odds of self-reporting dysmenorrhea (AOR, 1.73 (95% CI, 1.16–2.57)). Finally, a significant association between irregular menstrual cycle (*p* < 0.01), not lived with their parent (*p* < 0.04), and self-reported dysmenorrhea was found.

**Conclusion:**

This study establishes that dysmenorrhea is high among adolescents in Shai Osudoku District which negatively affects the daily activity of majority of them.

## 1. Introduction

Dysmenorrhea, also referred to as painful periods, is defined as pain during menstruation [1, 2] and accounted to be the most common reason why female adolescents visit gynecological clinics [3] in Ghana. Although dysmenorrhea is not life threatening, it can be painful for many adolescents. However, data on the prevalence and predictors of dysmenorrhea, its effect, and coping mechanisms among adolescent girl population of the Greater Accra Region are scanty.

This research is a contribution to existing theories and also identification of an ignored pattern in the previous literature. Again, this research provides insights to ensure that menstruation education addresses the social and psychological impact of the dysmenorrhea as well as the physical elements of menstruation.

Published studies revealed spasmodic rates of dysmenorrhea ranging from 16% to 91% [4, 5], with higher rates reported in adolescent populations [6]. Furthermore, the prevalence rate is estimated to be 85% in the United States of America [7], 84.1% in Italy [8], and 40.7% in India [9]. Besides, it is problematic to explain the differences in the prevalence of dysmenorrhea. Nevertheless, the use of different measure for the definition of dysmenorrhea, individual perceptions of pain, but not limited to diverse study populations could be the reasons for such variations.

There are few studies on the prevalence of dysmenorrhea among adolescents published in Ghana. A cross-sectional study conducted in Northern Ghana with a sample of 293 subjects revealed that about 83.6% of university students had dysmenorrhea [10]. Another study conducted among female senior secondary school student, on Accra, Ghana, with a sample of 456 subjects showed that the prevalence of dysmenorrhea was 74.4% [11]. In addition, Aziato et al. in their qualitative study among students in Ghana with a sample of 16 subjects reported that participants employed exercise, water, and diet therapy, as well as the use of herbal medications in managing their dysmenorrhea [12]. Collectively, previous studies have shown the current understanding of prevalence rates of dysmenorrhea but fail to analysis predictors, its effects, and coping mechanisms extensively.

Considering insufficient information on dysmenorrhea in Ghana and the fact that individual studies may have inadequate statistical power because of sample size, we sought to provide cross-sectional data on the prevalence and predictors of dysmenorrhea, its effect, and coping mechanisms among adolescents in Shai Osudoku District and to provide the necessary reference data on dysmenorrhea predictive factors to drive its prevention and control strategies in this district.

## 2. Materials and Methods

### 2.1. Research Design and Setting

We conducted a cross-sectional study in September and November 2017 in some selected schools in Shai Osudoku District, Ghana. It is the largest district in the Greater Accra Region in terms of land size and found in the southeastern part of Ghana. Dodowa is the district capital, which is about 39 km from the regional capital. The total estimated population of the district is 60915.

### 2.2. Study Population

A total number of 760 healthy adolescents aged 12–19 years formed the study population. Purposeful sampling was adopted to select unmarried girls who had experienced menstruation and not given birth before. Again, who responded “don't know” or who did not respond to the questions completely were excluded.

### 2.3. Data Collection Procedure

#### 2.3.1. Assessment of Outcome and Definition

The outcome variable in our analysis was any history of dysmenorrhea. All participants who answered “yes” to the question “have you ever had a menstrual pain?” considered to have a history of dysmenorrhea.

#### 2.3.2. Assessment of Exposure Variables

The questionnaire regarding menstrual characteristics, its effects, and coping strategies consisted of open- and close-ended questions engaged in the data collection. The questionnaire pretested to assess for clarity and completeness of the questions. The questionnaire for exposure variables was divided into 3 parts. The first part inquired about the age, level of education, place of stay (with parents or without parents), and the respondent's menstrual characteristics which include category and responses. These are chronological age (12–15 or 16–19 years), age at menarche in years (<13, 13 to 15, and 16–19), the history of the menstrual cycle (irregular or regular cycles), the nature of menstrual flow (light or moderate or heavy), length of the cycle (<21 or ≥21 days), duration of menstruation in days (≤3 or >3), and family history of dysmenorrhea (yes/no).

The second part inquired about the respondents the effect of menstrual pain on quality of life which include pain intensity (mild, moderate, or severe pain), duration of menstrual pain in days (≤3 or >3), restriction in physical activities (yes/no), absenteeism (yes/no), social withdrawal (yes/no), and unnecessary irritation (yes/no).

The third part inquired about the respondents coping strategies they have adopted, which include mostly ignored menstrual pain (yes/no), self-medication (yes/no), physical exercise for lessening their menstrual pain (yes/no), most adopted home remedies (relaxation, hot application, and herbs), and finally whether respondents consult a physician for assistance (yes/no).

### 2.4. Statistical Analysis

We analyzed the data using the Statistical Package for the Social Sciences (SPSS) programme IBM version 20. We performed an exploratory analysis using the Pearson chi-square test to determine the association between exposure variables and the outcome variable. Multiple logistic regression was employed to estimate the association between chronological age, level of education, place of stay, age at menarche, the history of the menstrual cycle, the nature of the menstrual flow, length of the cycle, duration of menstruation, and family history of dysmenorrhea. Covariates considered as after adjustment variables included severe irregular menstrual cycle and respondents who do not live with their parents. Finally, for all odds ratios, we calculated a 95% confidence interval (CI). A statistical significance assumed at *p* < 0.05.

### 2.5. Ethical Considerations

The leadership at each school provided facility-level consent for participation and permission for the researchers to collect data on every eligible adolescent who volunteers to participate. We also obtained verbal informed consent before data collection. In addition, all the respondents who qualify informed about the study, its objectives, and method of data collection. The questionnaires were reviewed by the School of the Public Health, Adventist University of Africa, Kenya, and the School of Nursing, Valley View University, Ghana.

## 3. Results

A total of 760 healthy adolescents participated in the study. Out of these, 680 of the respondents were included in the final analysis. The overall prevalence of self-reported dysmenorrhea in this study was 68.1% (95% CI, 65.0–72.0). The majority of respondents with dysmenorrhea (70.8%) were between the ages of 16–19 years old. The mean age of the respondents was 16.7 ± 1.98 years. About three-quarters (76.2%) of the dysmenorrhea were senior high school students but no significance was found (*p* > 0.20). With regard to living, 73.9% of the respondents who had dysmenorrhea lived with their parents and was significant (*p* < 0.04). Furthermore, adolescents who experienced their first menstruation at age 13–15 years reported more to have experienced dysmenorrhea (59.8%) than their counterparts but no significant association was found (*p* > 0.20). However, the proportion of dysmenorrhea that had menstrual irregularity was 28.5% and as a result, significantly associated with dysmenorrhea (*p* < 0.02). Nevertheless, no significant association was found between menstrual flow (*p* > 0.48), length of the menstrual cycle (*p* > 0.15), duration of menstruation (*p* > 0.10), family history of menstrual difficult (*p* > 0.59), and dysmenorrhea, as shown in Table 1.

### 3.1. Predictors of Self-Reported Dysmenorrhea

Model-based estimates of the association between exposure variables and self-reported dysmenorrhea are listed in Table 2. After adjustment for chronological age, level of education, place of stay, age at menarche, the history of the menstrual cycle, the nature of menstrual flow, length of the cycle, duration of menstruation, and family history of dysmenorrhea, a significant association between irregular menstrual cycle (*p* < 0.01), not lived with their parent (*p* < 0.04), and self-reported dysmenorrhea was found. However, no significant association between self-reported dysmenorrhea and chronological age, level of education, age at menarche, the nature of the menstrual flow, length of the cycle, duration of menstruation, and family history of dysmenorrhea was observed. Besides, adolescents who do not live with their parent experienced a 53.1% increase in odds of self-reporting dysmenorrhea (AOR, 1.53 (95% CI, 1.02–2.30; *p* < 0.04)). Similarly, respondents who had irregular menstrual cycle experienced a 72.5% increase in odds of self-reporting dysmenorrhea (AOR, 1.73 (95% CI, 1.16–2.57; *p* < 0.01)).

### 3.2. Effect of Dysmenorrhea on Adolescents

Table 3 shows the summary of the effect of dysmenorrhea among the study population. The results showed that 34.8% of adolescents with dysmenorrhea reported a history of severe menstrual pain, and a significant association was found (*p* < 0.04). The pain lasts more than three days for the majority (63.7%) of respondents, but no significant association was found with dysmenorrhea (*p*=0.13). The presence of menstrual pain affects physical activities by 22.5% and is significantly associated with self-reported dysmenorrhea (*p* < 0.04). On the contrary, absenteeism (6.9%), experienced poor concentration in class (27.9%), decreased academic performance (31.1%), experienced social withdrawal or poor personal relationship (38.2%), and experienced unnecessary irritation (38.2%) found no significant with self-reported dysmenorrhea.

### 3.3. Coping Mechanisms and Experience of Dysmenorrhea

Respondents asked about coping methods they have adopted. A higher proportion of dysmenorrheic adolescents reported mostly ignored their menstrual pain (56.6%). Nevertheless, when they took action, they mostly relied on self-medication (34.6%), less on physical exercise (14.9%), relaxation (25.7%), hot application (11.4), and herbs (6.7%) for lessening their menstrual pain. In addition, few (19.4%) of them consulted a physician. Nevertheless, no significant association with dysmenorrhea was found, as shown in Table 4.

## 4. Discussion

The prevalence of dysmenorrhea established in this study was 68.1% (95% CI, 65.0–72.0) and lower than previous studies that reported 83.6% [10] and 74.4% [11] prevalence rates in the different regions of Ghana. However, the prevalence documented in our study was higher than the 40.7% reported among students of the medical college in Delhi, India [9], and 33.8% [13] of adolescents experienced dysmenorrhea. This variation may be due to the use of different measures for the definition of dysmenorrhea and individual perceptions of pain.

We compared adolescents with a history of the irregular menstrual cycle, those who do not live with their parents, and self-reported dysmenorrhea. After adjustment for potential confounders, we observed that history of the irregular menstrual cycle and adolescents who do not live with their parent were associated with significantly increased odds of self-reported dysmenorrhea.

Therefore, the current analysis is consistent with previous studies suggesting a link between menstrual irregularity and dysmenorrhea. Nohara et al. discovered the relationship between irregular menstrual cycles and stress, the smell of cigarettes, age, smoking habits, and dysmenorrhea [14, 15]. We also found that 28.5% of adolescents with dysmenorrhea reported irregular menstrual cycle. Previous studies have reported that the prevalence of menstrual irregularity ranges from 10% to 38% in menstruating women, which is consistent with our findings [16, 17]. The significant association between irregular menstrual cycle and dysmenorrhea that we found does not imply a causal relationship. Certainly, it is possible that one or more mechanisms associated with both irregular menstrual cycle and dysmenorrhea may be responsible for the development of both. First, the major factor associated with dysmenorrhea is the increased production of prostaglandins by the endometrium, which are released during the menstrual flow causing uterine contractions and pain [18]. In addition, vasopressin also increases uterine contractility and causes ischemic pain as a result of vasoconstriction [18].

The study revealed that 59.8% of the respondents experienced menarche between the ages of 13–15 years had dysmenorrhea. This finding supports previous studies, which reported that early menarche was more likely to experience dysmenorrhea [19, 20]. However, no significant association was found between age at menarche and experience of dysmenorrhea (*p* > 0.20). Again, the 11.0% prevalence of dysmenorrhea among the respondents experiencing menarche between the age >15 year supports the previous studies which reported that early menarche associated with an increase in primary dysmenorrhea [21, 22]. Therefore, reproductive health education should address the social and psychological impact of the menarche as well as the physical elements of menstruation.

More so, previous studies have suggested an association between restriction of physical activities and experience of dysmenorrhea [23, 24] which supports our findings. Our finding revealed that 22.5% of adolescents with dysmenorrhea reported a history of physical activity restriction during menstruation. This percentage was similar to the previous study done in Mexican high school students, which reported a 24.1% physical activity restriction [25]. The prevalence rate presents evidence for the progressing education of dysmenorrhea as a public health problem in this age group.

In our study, 6.9% of adolescents with dysmenorrhea reported absenteeism. Previous studies have reported that 5% to 14% were absent from school as a result of dysmenorrhea [26]. Again, previous studies have reported that dysmenorrhea is the leading cause of recurrent short-term school absence [19, 27, 28]. Our finding was similar to 9.2% absenteeism reported in Ghana [11] and lower than 44.0% in Sri Lanka, where young women reported missing classes. These difference rates may be associated with the variability in cultural perceptions and responses to pain [20].

Furthermore, 27.9% of adolescents with dysmenorrhea reported poor concentration during classes. The disturbing part was that it decreased academic performance by 31.1% of the respondents. These findings are consistent with earlier studies, which reported that menstrual pain causes females the loss of concentration and the decrease in school performance and routine work [9, 17]. Therefore, these results could have a negative effect on the adolescent's psychosocial and cognitive behavior [29].

Again, 38.2% of adolescents with dysmenorrhea reported social withdrawal or poor personal relationship. This affects their mood [30], thereby leading to attitudinal changes within the family and among friends [31]. The findings support previous studies which documented that menstrual pain at times accompanied by physical pain and might finally cause physical, emotional, and social negative effects [20, 32].

Moreover, 56.6% of adolescents with dysmenorrhea reported that they mostly ignored their menstrual pain. However, when they took action, they chiefly relied on self-medication (34.6%). The finding supports the previous study, which reported that the treatment of dysmenorrhea includes over the counter drug such as ibuprofen [33].

Several research results suggested that most adolescents with dysmenorrhea do not consult a doctor [6, 34]. Our finding was not different, which showed only 19.4% of adolescents with dysmenorrhea reported to have consulted a physician for their menstrual pain, which highlights its importance as a public health issue. For that reason, most adolescents choose to use home remedies or nonpharmacological remedies for their pain [35]. The finding also showed that 14.9% of the adolescents engage in some form of physical exercise, although previous studies suggested that the role of physical activity in reducing dysmenorrhea and its pain intensity found a little effect [36]. However, there is epidemiological evidence that suggests dysmenorrhea occurs less often in those who exercise regularly [37, 38].

Finally, an earlier study reported that the treatment of dysmenorrhea includes but not limited to the use of a heating pad [33], which supports our finding where 11.4% of the dysmenorrheic adolescents use a hot application. Besides, 6.7% of the adolescents use herbs for lessening their menstrual pain. These findings support the previous studies which reported that more than one-third of females used nonpharmacological pain relief methods than pharmacological methods [4, 39]. Therefore, health education about nonpharmacological pain relief methods for the treatment of dysmenorrhea is urgently required.

## 5. Limitation of the Study

Despite the careful design of the study, limitation remains. The study selection involved the schools in the district capital only, which potentially limits the generalization of findings to the entire population.

## 6. Conclusion

The high prevalence of dysmenorrhea established reveals the magnitude of the problem in Shai Osudoku District. The adolescent who does not live with their parent and irregular menstrual cycle were significantly associated with dysmenorrhea. Furthermore, the consequence of untreated dysmenorrhea includes but not limited to social withdrawal, school absenteeism, having poor concentration, a decrease in academic performance, and restriction in daily activities. Adolescents with dysmenorrhea also had a negative attitude consulting a physician, highlighting its importance as a public health issue.

## Figures and Tables

**Table 1:** Univariate analysis for the experience of dysmenorrhea among the study participants.

Variables	Dysmenorrhea		Total (%)	*p* value
	No (%)	Yes (%)		
*Age in years*				
12–15	66 (30.4)	135 (29.2)	201 (29.6)	>0.74
16–19	151 (69.6)	328 (70.8)	479 (70.4)	
*Level of education*				
Junior high school	64 (29.5)	148 (32.0)	212 (31.2)	>0.20
Senior high school	153 (70.5)	315 (68.0)	468 (68.8)	
*Place of stay*				
Not lived with parents	41 (18.9)	121 (26.1)	162 (23.8)	**<0.04**
Lives with parents	176 (81.1)	342 (73.9)	518 (76.2)	
*Age at menarche*				
<13 years	74 (34.1)	135 (29.2)	209 (30.7)	>0.20
13–15 years	114 (52.5)	277 (59.8)	391 (57.5)	
>15 years	29 (13.4)	51 (11.0)	80 (11.8)	
*History of the menstrual cycle*				
Irregular cycles	43 (19.8)	132 (28.5)	175 (25.7)	**<0.02**
Regular cycles	174 (80.2)	331 (71.5)	505 (74.3)	
*Nature of menstrual flow*				
Light flow	54 (24.9)	96 (20.7)	150 (22.1)	>0.48
Moderate flow	148 (68.2)	333 (71.9)	481 (70.7)	
Heavy flow	15 (6.9)	34 (7.3)	49 (7.2)	
*Length of cycle*				
<21 days	24 (11.1)	70 (15.1)	94 (13.8)	>0.15
≥21 days	193 (88.9)	393 (84.9)	586 (86.2)	
*Duration of menstruation*				
<3 days	14 (6.5)	48 (10.4)	62 (9.1)	>0.10
≥3 days	203 (93.5)	415 (89.6)	618 (90.9)	
*A family history of dysmenorrhea*				
No	159 (73.3)	330 (71.3)	489 (71.9)	>0.59
Yes	58 (26.7)	133 (28.7)	191 (28.1)	

**Table 2:** Multiple logistic regression results modeling self-reported dysmenorrhea.

Variables	Dysmenorrhea	
	AOR (95% CI)	*p* value
*Age in years*		
12–15	1	—
16–19	1.25 (0.76–2.05)	>0.37
*Level of education*		
JHS	1	—
SHS	0.75 (0.46–1.22)	>0.25
*Place of stay*		
Not lived with their parents	1.53 (1.02–2.30)	**<0.04**
Lives with their parents	1	—
*Age at menarche*		
<13 years	1.03 (0.59–1.78)	>0.92
13–15 years	1.41 (0.84–2.37)	>0.19
>15 years	1	—
*History of menstrual cycle*		
Regular cycle	1	—
Irregular cycle	1.73 (1.16–2.57)	**<0.01**
*Nature of menstrual flow*		
Light flow	1	—
Moderate flow	1.27 (0.85–1.89)	>0.24
Heavy flow	1.21 (0.59–2.45)	>0.61
*Length of cycle*		
<21 days	1.13 (0.55–2.32)	>0.75
≥21 days	1	—
*Duration of menstruation*		
<3 days	1.61 (0.66–3.94)	>0.30
≥3 days	1	
*Family history of dysmenorrhea*		
No	1	—
Yes	1.10 (0.76–1.60)	>0.60

**Table 3:** Univariate analysis of the effect of dysmenorrhea among the study participants.

Variable	Dysmenorrhea		Total (%)	*p* value
	No (%)	Yes (%)		
*Pain intensity*				
Mild pain	87 (40.1)	154 (33.3)	241 (35.4)	**<0.04**
Moderate pain	75 (34.6)	148 (32.0)	223 (32.8)	
Severe pain	55 (25.3)	161 (34.8)	216 (31.8)	
*Duration of the pain*				
≤3 days	66 (30.4)	168 (36.3)	234 (34.4)	>0.13
>3 days	151 (69.6)	295 (63.7)	446 (65.6)	
*Restriction in physical activities*				
No	183 (84.3)	359 (77.5)	542 (79.7)	**<0.04**
Yes	34 (15.7)	104 (22.5)	138 (20.3)	
*Absenteeism*				
No	206 (94.9)	431 (93.1)	637 (93.7)	>0.36
Yes	11 (5.1)	32 (6.9)	43 (6.3)	
*Poor concentration*				
No	161 (74.2)	334 (72.1)	495 (72.8)	>0.58
Yes	56 (25.8)	129 (27.9)	185 (27.2)	
*Decreased academic performance*				
No	142 (65.4)	319 (68.9)	461 (67.8)	>0.37
Yes	75 (34.6)	144 (31.1)	219 (32.2)	
*Social withdrawal*				
No	191 (88.0)	400 (86.4)	591 (86.9)	>0.56
Yes	26 (12.0)	63 (13.6)	89 (13.1)	
*Unnecessary irritation (mood swing)*				
No	123 (56.7)	286 (61.8)	409 (60.1)	>0.21
Yes	94 (43.3)	177 (38.2)	271 (39.9)	

**Table 4:** Prevalence of mostly ignore the pain, practices self-medication, engages in physical exercise, use home remedies to reduce the pain, and consult a physician based on dysmenorrhea.

Variables	Dysmenorrhea		Total (%)	*p* value
	No (%)	Yes (%)		
*Mostly ignore the pain*				
No	93 (42.9)	201 (43.4)	294 (43.2)	>0.89
Yes	124 (57.1)	262 (56.6)	386 (56.8)	
*Practices self-medication*				
No	149 (68.7)	303 (65.4)	452 (66.5)	>0.41
Yes	68 (31.3)	160 (34.6)	228 (33.5)	
*Engages in physical exercise*				
No	193 (88.9)	394 (85.1)	587 (86.3)	>0.17
Yes	24 (11.1)	69 (14.9)	93 (13.7)	
*Home remedies used to reduce the pain*				
None	131 (60.4)	260 (56.2)	391 (57.5)	>0.19
Rest	58 (26.7)	119 (25.7)	177 (26.0)	
Hot application	19 (8.8)	53 (11.4)	72 (10.6)	
Herbs	9 (4.1)	31 (6.7)	40 (5.9)	
*Consult a physician*				
No	182 (83.9)	373 (80.6)	555 (81.6)	>0.30
Yes	35 (16.1)	90 (19.4)	125 (18.4)	

## Data Availability

The data used to support the findings of this study are included within the article.
